# Editorial: Advances in physical and psychosocial telecare: promises and pitfalls

**DOI:** 10.3389/fresc.2024.1490739

**Published:** 2024-09-13

**Authors:** Błażej Cieślik, Justyna Mazurek, Robert Gajda, Joanna Szczepańska-Gieracha

**Affiliations:** ^1^ Healthcare Innovation Technology Lab, IRCCS San Camillo Hospital, Venice, Italy; ^2^ University Rehabilitation Centre, Wroclaw Medical University, Wroclaw, Poland; ^3^ Collegium Medicum, Jan Dlugosz University in Częstochowa, Częstochowa, Poland; ^4^ Center for Sports Cardiology at the Gajda-Med Medical Center in Pultusk, Pultusk, Poland; ^5^ Faculty of Physiotherapy, Wroclaw University of Health and Sport Sciences, Wroclaw, Poland

**Keywords:** digital health interventions, home-based rehabilitation, remote support, telehealth, telemedicine, telepractice, telerehabilitation, information and communication technology

**Editorial on the Research Topic**
Advances in physical and psychosocial telecare: promises and pitfalls

Telehealth, telecare, and telemedicine are terms that are often used interchangeably, yet they represent distinct concepts within healthcare delivery. Although these terms differ in their specificity, they all encompass the provision of health-related services at a distance using information and communication technology (ICT) (1). Telecare encompasses the use of digital technologies such as smartphones, tablets, computers for virtual consultations, wearable devices for monitoring health metrics, and digital tools for remote diagnostics (1). These devices can be augmented with advanced ICT technologies, such as artificial intelligence (AI), Internet of Things (IoT), and extended reality (XR), which includes virtual reality (VR), augmented reality (AR), and mixed reality (MR) to enhance healthcare delivery and patient engagement (2–4). Telecare often utilizes asynchronous methods, such as store-and-forward techniques via secure messaging and email, along with wearable devices and activity trackers that introduce a delay between the transmission and reception of health information (5). In contrast, synchronous telecare involves real-time communication technologies like telephone and videoconferencing, which facilitate instantaneous information exchange between users (5).

**Figure 1:**
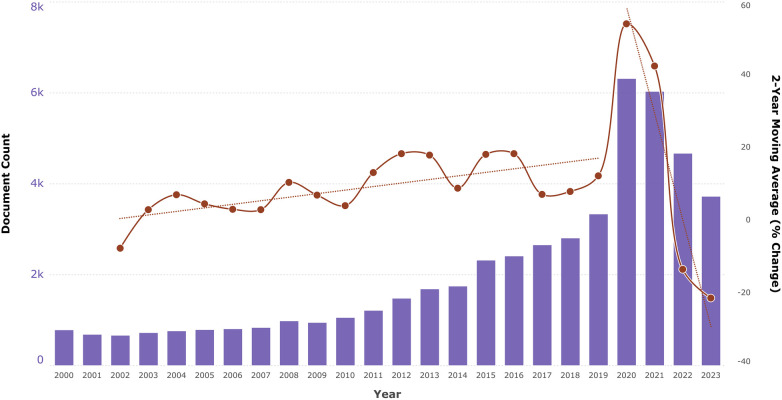
Number of documents indexed under the telemedicine [MESH] category in MEDLINE (via pubMed) from 2000 to 2023, showing the 2-year moving average percentage change and corresponding trend lines.

The rise in telecommunication-based studies in recent years reflects the growing significance of these technologies in healthcare. As illustrated in Figure 1, the number of documents included in the Telemedicine [MESH] category of MEDLINE (via PubMed) has steadily increased, with a notable spike in 2020 due to the COVID-19 pandemic and the urgent need for remote patient support. This spike was followed by a period of normalization from 2021 to 2023, suggesting that while interest in telecare surged during the pandemic, a more measured approach to its implementation is now being taken. This trend may also indicate that some researchers used the “*tele”* prefix as a buzzword to capitalize on pandemic-driven interest (6).

This Research Topic aims to highlight interdisciplinary research approaches that address knowledge gaps and generate evidence for the use of telecare in various rehabilitation fields, collectively referred to as telerehabilitation—a subset of telehealth specifically focused on providing rehabilitation services remotely using ICT. This collection includes four articles: two reviews focused on home-based interventions, one observational study examining participant satisfaction with a telerehabilitation program for individuals with chronic conditions, and a study protocol proposing the development of a telephone follow-up scale for patients with disorders of consciousness. These studies collectively underscore the potential of technological solutions to enhance home-based rehabilitation and physical activity, particularly through increased training volume and flexibility, which are necessary for effective motor learning and physical activity management.

For example, Forman et al. categorize technological approaches into sensory stimuli training, digital exchange of information, and telerehabilitation, demonstrating how each can promote neuroplasticity and motor learning. Meanwhile, Stavric et al. show that self-guided digital interventions, especially those incorporating behavioral strategies like goal setting, feedback, and self-regulation, can significantly improve physical activity levels both immediately and over the long term. Despite these advances, both studies highlight existing gaps, such as the need for personalized and adaptive feedback, challenges in maintaining long-term engagement, and the limited accuracy of objective measurements for certain populations.

Evaluating intervention effectiveness also involves understanding patient satisfaction, as demonstrated by Roy et al. Their retrospective study explored the determinants of satisfaction among individuals with chronic conditions who participated in a telerehabilitation program during the COVID-19 pandemic. Findings indicated high overall satisfaction, with specific factors such as perceived benefit, adapted home exercises, and the convenience of staying home being the primary drivers. These insights underscore the importance of patient-centered design in telerehabilitation programs and suggest areas for further exploration, such as the role of therapists and the need for personalized approaches to enhance long-term outcomes.

Additionally, accurate outcome measures are vital for tracking patient recovery and informing treatment decisions. This is the focus of the study by Shou et al., who aim to develop a telephone follow-up scale tailored for individuals with disorders of consciousness to improve monitoring of their recovery in non-clinical settings. The study seeks to create a sensitive, straightforward tool that allows for remote assessment of consciousness, ensuring consistent and reliable data collection. While this tool shows promise for addressing current gaps in follow-up care, we must await the full results to fully assess its effectiveness and adaptability in diverse settings.

These studies demonstrate the significant potential of telehealth technologies to enhance healthcare delivery by increasing training volume, flexibility, and patient satisfaction, especially for individuals with neurological disabilities, chronic conditions, and disorders of consciousness. These findings align with previous research that supports the effectiveness of remote care in maintaining patient engagement and improving outcomes through tailored, home-based interventions (7, 8). However, important gaps remain, including the need for more personalized and adaptive feedback, better strategies for sustaining long-term engagement, and improved accuracy of objective measures in diverse populations. Additionally, the role of healthcare providers in remote settings needs further exploration to optimize patient outcomes and satisfaction. Lastly, barriers to the clinical adoption of telehealth, such as regulatory challenges, technology access, and the integration of telehealth into standard care practices, must be addressed to maximize its benefits (9, 10).
